# Anästhesieführung bei Patienten mit Dopa-responsiver Dystonie (Segawa-Syndrom): Darstellung der Pathophysiologie, Klinik und Vorgehensweise anhand zweier Fallberichte

**DOI:** 10.1007/s00101-020-00898-0

**Published:** 2020-12-08

**Authors:** K. Groß, S. Kleinschmidt

**Affiliations:** Klinik für Anaesthesiologie, Intensivmedizin und Schmerztherapie, Universitätsklinikum des Saarlandes, 66421 Homburg (Saar), Deutschland

**Keywords:** Bewegungsstörung, Levodopa, Perioperatives Management, Organfunktionseinschränkungen, Begleitmedikation, Movement disorders, Levodopa, Perioperative management, Impaired organ function, Concurrent medication

## Abstract

Die Dopa-responsive Dystonie (Segawa-Syndrom) ist eine sehr seltene neurologische Erkrankung. Bei korrekter Diagnosestellung und adäquater Therapie sind gravierende Organfunktionsstörungen kaum zu erwarten. Dennoch sind Kenntnisse über das Krankheitsbild und die möglichen Anästhesieverfahren bei der Versorgung dieser Patienten von Bedeutung. Anhand zweier Kasuistiken werden die wesentlichen Fakten zur perioperativen Betreuung dieser Patientengruppe dargestellt.

## Abstract (en)

Segawa syndrome (dopa-responsive dystonia [DRD]) is a rare neurometabolic disorder characterized by progressive dystonia, diurnal variation and tremors. It is caused by an enzymatic defect (a mutation of the *GTPCH1* gene located on chromosome 14q) in the synthesis of tetrahydrobiopterin, an important substrate for dopamine synthesis. In the case of early correct diagnosis, clinical symptoms are well-controlled by levodopa therapy. The disease has several features which may lead to organ dysfunctions (e.g. torticollis, scoliosis, dysphagia and immobilization), which may be of concern for the anesthesiologist. Presenting two case reports of female patients undergoing elective cesarean section and breast cancer surgery, the main principles of perioperative management are discussed. Either techniques of regional or general anesthesia can be performed safely. Preoperative medication with levodopa should not be interrupted. Pharmacological agents with an antidopaminergic mode of action have to be avoided as well as significant pain and emotional stress situations in the perioperative period. Surgery in an ambulatory setting may not be recommended.

## Einführung

Die autosomal-dominant vererbte Dopa-responsive Dystonie (DRD) ist eine seltene neurometabolische Erkrankung. Die Prävalenz wird auf etwa 0,5–1/1.000.000 Einwohner geschätzt [7, 8]. Die Erkrankung beginnt häufig im Vorschulalter mit einer Dystonie der unteren Extremitäten und einer Equinovarus-Haltung der Füße (Plantarflexion, Inversion der Ferse und Adduktionshaltung mit medialer Deviation), woraus Gangstörungen resultieren. Die Symptome, welche auch in eine generalisierte Dystonie münden können, weisen oft tagesabhängige Schwankungen im Schweregrad auf; so mildert sich die Symptomatik oft nach Schlafphasen ab [3, 7]. Auch durch körperliche und emotionale Belastung sind Verschlechterungen der Symptome möglich, sodass Patienten mitunter auch auf einen Rollstuhl angewiesen sein können.

Die Erkrankung wurde von Segawa et al. 1976 erstmals in einer englischsprachigen Zeitschrift beschrieben [4]. Pathophysiologisches Korrelat der Erkrankung sind Mutationen im *GCH1*-Gen auf dem Chromosom 14 (Genlocus: 14q22.1–q22.2). Dieses Gen kodiert die Synthese der GTP-Zyklohydrolase 1 (GTPCH1), welches an der Synthese des Tetrahydrobiopterins (THB) beteiligt ist. THB selbst ist ein essenzieller Kofaktor der Tyrosinhydroxylase und gleichzeitig ein geschwindigkeitsbestimmender Schritt der Dopaminsynthese [7]. Die Penetranz der autosomal-dominant vererbten Erkrankung ist unterschiedlich (Frauen sind häufiger betroffen als Männer), sodass die Familienanamnese nicht immer richtungweisende Befunde liefert. Zur Diagnose führt im Regelfall ein positiver Dopa-Test: Niedrige L‑Dopa-Dosierungen (schon ab 20 mg/Tag) zeigen eine beeindruckende und dauerhafte Besserung der klinischen Symptome [3]. Die Therapie muss lebenslang erfolgen. Dennoch ist es möglich, dass Patienten mit jahrelang unerkanntem Krankheitsbild und/oder unzureichender Therapie körperliche Funktionseinschränkungen als Sekundärfolge davontragen, welche für die präoperative Evaluation und für die Anästhesieführung von Relevanz sein können, so u. a. Skoliosen der Wirbelsäule, Torticollis oder Störungen des Schluckakts einschließlich einem gastroösophagealen Reflux (Tab. 1). Daher sind die Kenntnis der Erkrankung und das perioperative Management für den Anästhesisten von Bedeutung. Dies soll anhand zweier Fallberichte dargestellt werden.

**Table 1:** 

Begriff	Beschreibung
ICD-10 Code/Abkürzung	Code G24.1 (auch G24.8)/DYT5a
Synonyma	Hereditäre progressive Dystonie mit tageszeitlicher Fluktuation, GTPCH1-defiziente Dopa-responsive Dystonie
Erbgang	Autosomal-dominant mit variabler Penetranz, Frauen häufiger betroffen als Männer
Pathophysiologie	Defekt der GTPCH 1, Genlocus auf Chromosomenabschnitt 14q
Diagnostik	Klinisch: Positiver L‑Dopa Test Labor: erniedrigter Gesamt-THB-Spiegel im Liquor, molekulargenetische Diagnostik
Auswahl von Differenzialdiagnosen	Autosomal-rezessive DRD (DYT5b), Torsionsdystonie, myoklonische Dystonie, hereditäre spastische Paraplegie, Parkinsonismus im frühen Erwachsenenalter
Mögliche funktionelle Störungen	Skoliose, Torticollis, schwieriger Atemweg, Dysphagie, gastroösophagealer Reflux, Hypotension und orthostatische Dysregulation (mögliche Folge der L‑Dopa-Therapie)

*DYT* Dystonie, *GTPCH1* Guanosintriphosphat-Zyklohydrolase1, *THB* Tetrahydrobiopterin, *DRD* Dopa-responsive Dystonie

## Kasuistik 1

Eine 21-jährige normotensive Erstgravida stellte sich nach bis dato unauffälligem Schwangerschaftsverlauf am errechneten Termin zur primären Sectio caesarea vor. Fremdanamnestisch waren bei der Patientin erste Symptome beim freien Gehen im 12. Lebensmonat aufgefallen („Zehenspitzengang“) mit in der Folgezeit erheblichen Störungen der Koordination („zittriges Schriftbild, Krämpfe in den Händen“), die bei einer normalen geistigen Entwicklung einen geregelten Schulbesuch zunächst deutlich behinderten. Der Vater, die Großmutter väterlicherseits und eine Tante väterlicherseits litten ebenfalls an „Bewegungsstörungen“ und „Ballenhohlfüßen“. Die Diagnose „Segawa Syndrom“ wurde im 7. Lebensjahr gestellt und humangenetisch bzw. molekulargenetisch verifiziert (autosomal-dominanter Erbgang, heterozygoter Allelstatus, *GCH1*-Mutation 512 T > A). Unter der konsequenten medikamentösen Therapie (die aktuelle Medikation betrug 2‑mal 100/25 mg Dopa/Benserazid täglich) waren in der Folgezeit die Symptome deutlich rückläufig und eine normale altersentsprechende Entwicklung gewährleistet. Eine im Pubertätsalter auftretende „Antriebsarmut“ wurde temporär mit Citalopram (10–20 mg/Tag) therapiert und die oben genannte Medikation auch während der Schwangerschaft beibehalten.

Obwohl keine maternalen und fetalen Kontraindikationen für eine vaginale Entbindung bestanden, wurde von dem mitbehandelnden Neurologen und den Geburtshelfern eine primäre Schnittentbindung vorgeschlagen. Wesentlicher Grund für diese Entscheidung war die Annahme, dass Schmerzen und „Stress“ bei einem zeitlich nicht planbaren Geburtsverlauf Myoklonien und Dystonien triggern können. Die Patientin willigte in die vorgeschlagene Vorgehensweise ein. Die Anlage der Spinalanästhesie zur primären Sectio ohne anxiolytische Prämedikation mit 2,2 ml Bupivacain 0,5 % hyperbar und additiv 5 µg Sufentanil (Punktion L3/L4 mit einer 25-G-Sprotte-Nadel) war problemlos, die Anästhesie suffizient. Die Patientin wurde bei einem intraoperativen Blutverlust von ca. 500 ml von einem vitalen Neugeborenen entbunden. Der weitere Verlauf war unter rascher postoperativer Aufnahme der vorbestehenden Medikation und einer Schmerztherapie mit Ibuprofen (3-mal 600 mg/Tag) und Oxycodon (2-mal 5 mg/Tag) nach Abklingen der Spinalanästhesie unauffällig. Die Patientin wurde nach postoperativer Betreuung auf der Wachstation am 4. postoperativen Tag aus der stationären Behandlung entlassen. Die Nachsorgetermine wurden wahrgenommen und wiesen bei unveränderter Medikation auf einen stabilen neurologischen Befund hin.

## Kasuistik 2

Eine 56-jährige normgewichtige und normotensive Patientin stellte sich bei diagnostiziertem Mammakarzinom zur anästhesiologischen Voruntersuchung und Aufklärung vor. Für den Folgetag war eine brusterhaltende Resektion mit einer „Sentinel-node-Biopsie“ geplant. Anamnestisch unterzog sich die Patientin lediglich zweier Eingriffe zur Kataraktchirurgie, die in Retrobulbäranästhesie durchgeführt wurden. Die Patientin berichtete, im Alter von ca. 16 Jahren ein Zittern in der rechten Hand bemerkt zu haben. Später trat als zusätzliches Symptom ein „unwillkürliches Kopfrücken“ auf. Die Symptome wiesen tagesabhängige Schwankungen auf, waren nachts rückläufig und hätten sich in „Stresssituationen“ verstärkt. Ein erster Therapieversuch mit Ropinirol war erfolglos. Unter einer probatorischen Therapie mit L‑Dopa kam es dagegen zu einer dauerhaften Beschwerdebesserung. Die Diagnose „DRD, Segawa-Syndrom“ wurde schließlich im Alter von 37 Jahren gestellt und ein juveniler M. Parkinson differenzialdiagnostisch ausgeschlossen. Eine molekulargenetische Diagnostik erfolgte offenbar nicht, bzw. Befunde hierüber waren nicht verfügbar. Die Familienanamnese ergab keine richtungweisenden genetischen Auffälligkeiten. Rezidivierende depressive Episoden wurden mit Mirtazapin und Citalopram erfolgreich therapiert. Die Medikation (jeweils kumulierte Tagesdosis) zum Zeitpunkt der Anästhesie bestand aus Citalopram 20 mg, L‑Dopa/Benserazid 100/25 mg sowie L‑Dopa/Carbidopa 200/50 mg.

Die Anästhesieführung erfolgte ohne eine sedierende oder anxiolytische Prämedikation als Larynxmaskennarkose mit insgesamt 25 µg Sufentanil, 180 mg Propofol und Desfluran (1 MAC endtidal). Zur Schmerztherapie erhielt die Patientin 1,5 g Metamizol und kumulativ 7 mg Piritramid am Operationstag. Zur PONV-Prophylaxe wurden Dexamethason und Ondansetron (jeweils 4 mg) intraoperativ verabreicht. Die präoperativ bestehende Medikation wurde unmittelbar postoperativ auf der Wachstation wiederaufgenommen. Die Patientin wurde am 2. postoperativen Tag nach unauffälligem Verlauf in die ambulante Weiterbehandlung entlassen.

## Diskussion

Die beschriebene niedrige Prävalenz der DRD spiegelt sich in der vorhandenen Literatur wider. Neben Einzelkasuistiken [5, 9] konnten Howze et al. [1] an der Mayo-Klinik Rochester über einen Zeitraum von 12 Jahren insgesamt 12 Patienten im Alter von 3 bis 77 Jahren mit DRD identifizieren, die sich insgesamt 25 Prozeduren mit anästhesiologischer Beteiligung unterzogen. Es fanden sich bei der Heterogenität der Patientengruppe bezüglich Alter, Medikation, Komorbiditäten und der operativen Eingriffe keine anästhesiebedingten Komplikationen. So wurden u. a. Muskelrelaxanzien und volatile Anästhetika problemlos angewendet.

In den beiden vorliegenden Fällen waren die Patientinnen sehr gut über ihr Krankheitsbild informiert und achteten genau auf die korrekte Einnahme der Medikation. Beide Patientinnen erhielten zur Therapie einer Begleitdepression Citalopram, welches jedoch die Therapie der DRD mit Levodopa offenbar nicht beeinträchtigte. Dies lässt sich durch den Wirkmechanismus von Citalopram als selektivem Serotonin-Reuptake-Inhibitor erklären. Über die Weitergabe von L‑Dopa in Kombination mit Decarboxylasehemmern während der Schwangerschaft liegen unterschiedliche Berichte vor. Eine teratogene Wirkung beim Menschen ist bisher nicht sicher belegt [6]. In der publizierten Kasuistik von Sinha et al. [5] pausierte die Patientin mit DRD während der Schwangerschaft die Medikation mit L‑Dopa unter Inkaufnahme erheblicher Nebenwirkungen (Dystonien, „Rollstuhlpflichtigkeit“ im 3. Trimenon). Die Anästhesie zur primären Sectio erfolgte hier als CSE und war suffizient. Im vorliegenden Fall wurde aus fetaler Indikation auf eine medikamentöse Prämedikation mit Benzodiazepinen verzichtet. Ein einfühlsames Vorgehen mit Schaffung einer beruhigenden Atmosphäre war angezeigt und führte zu keiner Verschlechterung neurologischer Symptome im perioperativen Verlauf.

Obwohl in einer Kasuistik eine komplette Remission anatomischer Fehlstellungen (Skoliose, Torticollis) nach einem Krankheitsverlauf von 34 Jahren bis zur Diagnosestellung durch die Dopa-Therapie beschrieben wurde [2], sind aus anästhesiologischer Sicht mögliche bleibende körperliche Funktionsstörungen zu beachten. Direkte Organfunktionsstörungen im Sinne einer häufigen Komorbidität (z. B. Herzinsuffizienz, Niereninsuffizienz) durch die DRD selbst sind bisher nicht beschrieben. Als mögliche Folge der Therapie mit L‑Dopa kann es zu hypotensiven bzw. orthostatischen Episoden kommen. Als Ursache vermutet man die Verdrängung physiologischer postsynaptischer Neurotransmitter [5], vergleichbar dem Wirkmechanismus von α‑Methyldopa als Antihypertensivum. Eine Hypotension lag bei beiden Patientinnen nicht vor. Bei „fixierten“ Skoliosen nach langjährigem unerkannten Krankheitsverlauf können sich Einschränkungen der Lungenfunktion im Sinne einer restriktiven Funktionsstörung manifestieren, die bei der Anästhesieführung zu beachten sind. Aufgrund der Seltenheit der DRD sind keine Verlaufsbeobachtungen oder systematische Daten verfügbar.

Folgende Empfehlungen können zur Anästhesieführung bei Patienten mit DRD gegeben werden:

- Muskelrelaxanzien sind unter neuromuskulärem Monitoring sicher anwendbar, jedoch soll bei einer dauerhaften Immobilität die Gabe von Succinylcholin unterbleiben [9].
- Die in der klinischen Praxis verwendeten Induktionshypnotika und volatilen Anästhetika sind sicher anwendbar [1].
- Weiterhin sind Prädiktoren für einen schwierigen Atemweg oder ein Aspirationsrisiko zu erfassen und leitliniengerechte Vorkehrungen zu treffen.
- Aus pharmakologischen Überlegungen sind antidopaminerg wirkende Substanzen wie Metoclopramid oder Droperidol zu vermeiden. Hingegen erscheinen 5‑HT_3_-Antagonisten („Setrone“) zur antiemetischen Prophylaxe geeignet.
- Unterbrechungen der L‑Dopa-Therapie sind auf ein Minimum im perioperativen Verlauf zu beschränken.
- Aufgrund der Unwägbarkeiten des Krankheitsverlaufs in der postoperativen Phase sollte ein ambulantes Vorgehen bei Patienten mit DRD nicht erfolgen [8].

Beide Fallberichte zeigen, dass Regional- und Allgemeinanästhesieverfahren unter adäquater Vorbereitung bei Patienten mit DRD sicher angewendet werden können. Da bei der DRD der Muskelmetabolismus nicht primär betroffen ist, besteht *keine* Assoziation zur malignen Hyperthermie.

## Fazit für die Praxis

- Die Dopa-responsive Dystonie (DRD, Segawa-Syndrom) ist eine seltene hereditäre Erkrankung mit einer Inzidenz von etwa 0,5–1:1.000.000 Einwohner.
- Die Diagnose wird oft bereits im Kindesalter oder auch erst verzögert im Erwachsenenalter gestellt.
- Bei einer konsequenten Therapie mit L‑Dopa finden sich selten bleibende funktionelle Schädigungen, die zu Schwierigkeiten bei der anästhesiologischen Versorgung führen können.
- Es sind grundsätzlich alle Verfahren der Regional- und Allgemeinanästhesie durchführbar. Nur wenige Pharmaka sollten vermieden werden.
- Emotionale und/oder körperliche Belastungssituationen sind perioperativ zu vermeiden.

## Abkürzungen

A: Adenin
CSE: „Combined spinal epidural anaesthesia“
DRD: Dopa-responsive Dystonie
DYT: Dystonie
GTPCH1: Guanosintriphosphat-Zyklohydrolase 1
ICD: International Classification of Diseases
MAC: Minimale alveoläre Konzentration
PONV: Postoperative Übelkeit und Erbrechen
T: Thymin
THB: Tetrahydrobiopterin
