# Erratum zu: Anästhesieführung bei Patienten mit Dopa-responsiver Dystonie (Segawa-Syndrom): Darstellung der Pathophysiologie, Klinik und Vorgehensweise anhand zweier Fallberichte

**DOI:** 10.1007/s00101-021-00993-w

**Published:** 2021-06-30

**Authors:** K. Groß, S. Kleinschmidt

**Affiliations:** Klinik für Anaesthesiologie, Intensivmedizin und Schmerztherapie, Universitätsklinikum des Saarlandes, 66421 Homburg (Saar), Deutschland

**Erratum zu:**

**Anaesthesist 2020**

10.1007/s00101-020-00898-0

Der Artikel „Anästhesieführung bei Patienten mit Dopa-responsiver Dystonie (Segawa-Syndrom). Darstellung der Pathophysiologie, Klinik und Vorgehensweise anhand zweier Fallberichte“ von K. Groß und S. Kleinschmidt wurde ursprünglich Online First ohne „Open Access“ auf der Internetplattform des Verlags publiziert. Nach der Veröffentlichung in Bd. 70, Heft 4, S. 316–319 hatten sich die Autoren für eine „Open Access“-Veröffentlichung entschieden. Das Urheberrecht des Artikels wurde deshalb in © The Author(s) 2020 geändert.

